# Selecting molecules with diverse structures and properties by maximizing submodular functions of descriptors learned with graph neural networks

**DOI:** 10.1038/s41598-022-04967-9

**Published:** 2022-01-21

**Authors:** Tomohiro Nakamura, Shinsaku Sakaue, Kaito Fujii, Yu Harabuchi, Satoshi Maeda, Satoru Iwata

**Affiliations:** 1 Department of Mathematical Informatics, The University of Tokyo, Hongo 7-3-1, Bunkyo-ku, Tokyo 113-8656 Japan; 2 National Institute of Informatics, Hitotsubashi 2-1-2, Chiyoda-ku, Tokyo 101-8430 Japan; 3 Department of Chemistry, Faculty of Science, Hokkaido University, Kita 10 Nishi 8, Kita-ku, Sapporo, Hokkaido 060-0810 Japan; 4 Institute for Chemical Reaction Design and Discovery (WPI-ICReDD), Hokkaido University, Kita 21 Nishi 10, Kita-ku, Sapporo, Hokkaido 001-0021 Japan; 5 National Institute for Materials Science (NIMS), Research and Services Division of Materials Data and Integrated System (MaDIS), Tsukuba, Ibaraki 305-0044 Japan; 6 JST, ERATO Maeda Artificial Intelligence for Chemical Reaction Design and Discovery Project, Kita 10 Nishi 8, Kita-ku, Sapporo, Hokkaido 060-0810 Japan

**Keywords:** Cheminformatics, Applied mathematics, Computational science

## Abstract

Selecting diverse molecules from unexplored areas of chemical space is one of the most important tasks for discovering novel molecules and reactions. This paper proposes a new approach for selecting a subset of diverse molecules from a given molecular list by using two existing techniques studied in machine learning and mathematical optimization: graph neural networks (GNNs) for learning vector representation of molecules and a diverse-selection framework called submodular function maximization. Our method, called SubMo-GNN, first trains a GNN with property prediction tasks, and then the trained GNN transforms molecular graphs into molecular vectors, which capture both properties and structures of molecules. Finally, to obtain a subset of diverse molecules, we define a submodular function, which quantifies the diversity of molecular vectors, and find a subset of molecular vectors with a large submodular function value. This can be done efficiently by using the greedy algorithm, and the diversity of selected molecules measured by the submodular function value is mathematically guaranteed to be at least 63% of that of an optimal selection. We also introduce a new evaluation criterion to measure the diversity of selected molecules based on molecular properties. Computational experiments confirm that our SubMo-GNN successfully selects diverse molecules from the QM9 dataset regarding the property-based criterion, while performing comparably to existing methods regarding standard structure-based criteria. We also demonstrate that SubMo-GNN with a GNN trained on the QM9 dataset can select diverse molecules even from other MoleculeNet datasets whose domains are different from the QM9 dataset. The proposed method enables researchers to obtain diverse sets of molecules for discovering new molecules and novel chemical reactions, and the proposed diversity criterion is useful for discussing the diversity of molecular libraries from a new property-based perspective.

## Introduction

Chemical space^1–4^, a concept to represent an ensemble of chemical species, was originally established in medicinal chemistry^2,5^ and is used in a wide area of chemistry. The size of chemical space, i.e., the number of molecules in it, is estimated to be $10^{60}$ even if it is limited to drug-like molecules^6^, and other estimations of chemical-space sizes have also been reported^4,7^. In any case, the number of molecules is too large to explore exhaustively. Currently, more than 68 million molecules are registered in the chemical abstracts service (CAS) of Americal Chemical Society^8,9^, and some accessible online molecular databases, e.g., PubChem^10^, ZINC^11^, have been constructed. Moreover, owing to recent advances in high throughput screening, chemoinformatics^12^, and machine learning^13^, many chemical compounds have been discovered from chemical space in the fields of organic light-emitting diode^14^, organic synthesis^15^, and catalyst^16^. These are, however, small fractions of chemical space, and vast areas remain unexplored.

Selecting diverse molecules from chemical space is an important task for discovering molecules that exhibit novel properties and new chemical reactions^3,17^. In medicinal chemistry, diversity selection algorithms have been widely studied for exploring chemical space and discovering bioactive molecules^5,18–21^. The diversity of a set of molecules is also essential in molecular library design^17,22^. Furthermore, when analyzing the quality of molecular libraries, the way to assess their diversity is crucial. This paper contributes to diverse molecular selection by proposing a novel selection framework and a new criterion for evaluating the diversity of molecules.

For computing the diversity of sets of molecules, most existing methods start by specifying molecular descriptors, which encode molecules as vectors. Examples of molecular descriptors include ECFP^23^, MACCS keys^24^, and Daylight fingerprints^25^, which typically encode structural information of molecules as binary vectors. Given such descriptors, pairwise dissimilarities are defined to quantify how dissimilar two molecules are. A widely used pairwise dissimilarity is the Tanimoto coefficient (more precisely, the Tanimoto coefficient indicates a similarity value, and subtracting it from 1 yields the dissimilarity)^26^. Computation of molecular similarities constitutes a broad research area, and other approaches based on, e.g., graph edit distances^27^, cosine similarities of SMILES kernels^28^, maximum common substructures^29^, a root mean square deviation of 3D-molecular structures^30^ and the persistent homology (a topological signature)^31^ have also been proposed. Given such pairwise (dis)similarity measures, the diversity of sets of molecules is usually evaluated with, e.g., the mean pairwise dissimilarity (MPD) or the mean distance to closest neighbors calculated over selected molecules.

While the diversity of molecules can be computed as above, selecting molecules that maximize a diversity measure from given molecular lists is computationally more challenging. For example, a naive brute force search for selecting 10 out of 100 compounds requires calculating diversity values $$\left( {\begin{array}{l}100\\ 10\end{array}}\right) $$ times. To overcome this computational difficulty, the greedy algorithm, which iteratively selects a new molecule that is the most dissimilar to a set of currently selected molecules, has been widely used as an efficient heuristic method^32^. In each iteration, the dissimilarity between a new molecule and a set of selected molecules is computed according to a certain rule, e.g., MaxSum^33^ and MaxMin^34,35^, and the choice of such rules affects outputs of the greedy algorithm. The diversity of molecular sets obtained by the greedy algorithm is usually evaluated with, e.g., the MPD defined with the Tanimoto coefficient of MACCS keys. Thus calculated diversity values intrinsically depend on the choice of molecular descriptors and pairwise dissimilarities. Consequently, the existing framework for selecting molecules and evaluating the diversity puts much weight on structural information of molecules since molecular descriptors usually encode structural information of molecules and pairwise dissimilarities are calculated based on such structure-based descriptors.

On the other hand, exploration of chemical space that takes the diversity of molecular properties into account has been reported to be effective for discovering novel functional materials^36^. Also, in drug discovery, the Fréchet ChemNet Distance (FCD), which is a novel property-based metric using hidden layers of prediction models for bioactivities as representation of molecules, has been reported to be useful for evaluating models for generating molecules^37^. When it comes to discovering novel reactions, examining collections of molecules that are diverse regarding molecular properties (in particular, reactivity) is vital for efficient exploration of chemical space. Therefore, utilizing not only structural information but also properties of molecules can be a promising approach to pushing the diverse molecular selection framework to the next level, which will facilitate the discovery of novel molecules and new chemical reactions.

In the field of machine learning, neural network (NN) architectures have yielded great success in various areas such as image recognition and natural language processing. Following the achievements, researchers have applied them to molecular property prediction tasks. Among such approaches, graph neural networks (GNNs) have been gaining attention since many GNN-based prediction methods have achieved high performances^38–43^. GNNs transform molecular graphs into vectors, which are used in downstream property prediction tasks. Notably, GNNs generate vectors taking both molecular properties and structural information of molecules into account, and it is reported that molecular vectors obtained from trained GNNs successfully reflect chemists’ intuition of molecular structures^41^. Therefore, GNN-based molecular vectors can be effective alternatives to the aforementioned traditional molecular descriptors. However, to leverage GNN-based vectors for selecting diverse molecules, we need to discuss how to select diverse molecular vectors generated by GNNs, for which the existing structure-based selection framework is not necessarily appropriate.

Mathematically, the problem of selecting diverse items (in our case, molecular vectors) has been widely studied as *submodular function maximization*^44,45^. This framework is one of the best ways for diverse selection due to the following two advantages. First, many submodular functions for quantifying the diversity have been developed in various fields, and thus we can choose an appropriate one to achieve desirable diverse selection. In particular, some submodular functions can represent relationships between multiple molecules that pairwise similarities cannot capture. For example, the log-determinant function, a submodular function our method will use, serves as a volumetric diversity measure of molecular vectors. Such functions can offer us the potential for going beyond the existing pairwise-similarity-based framework. Second, and more importantly, we can mathematically guarantee the greedy algorithm to select near-optimally diverse molecules in terms of submodular function values. Specifically, resulting submodular function values are guaranteed to be at least $63\%$ of those achieved by optimal selection^44^. Moreover, the empirical performance of the greedy algorithm for submodular function maximization is known to be much better; it often achieves more than $90\%$ of optimal values^46,47^. Therefore, the submodularity-based approach enables us to efficiently obtain near-optimally diverse sets of molecules without relying on costly selection algorithms such as the brute force search.

This paper proposes a new approach to diverse molecular selection by utilizing the aforementioned GNN-based molecular vectors and the existing submodularity-based selection method. First, we train a GNN with property prediction tasks and use the trained GNN to transform molecular graphs into molecular vectors. Then, we define a submodular function that quantifies the diversity of those molecular vectors as volumes of parallelotopes spanned by them. Owing to the submodularity of the function, we can select near-optimally diverse molecular vectors by using the greedy algorithm. Both GNNs and submodular function maximization are known to be effective in various tasks, and thus each of them has been well studied. However, few existing studies utilize both of them for a single purpose. The only exception is a recent study on multi-robot action selection^48^, which uses GNNs in selection methods, while we use GNNs to design submodular functions. In view of this, our work provides a new type of application combining GNNs and submodular function maximization. Furthermore, to evaluate the diversity of selected molecules based on molecular property values, we introduce a new diversity measure using the Wasserstein distance^49,50^ to uniform distributions defined on molecular property values. This property-based measure can play a complementary role to the existing structure-based measures such as the MPD of the Tanimoto coefficients, thus enabling researchers to more profoundly discuss the diversity of molecules. Computational experiments compare the proposed method with the existing structure-based methods and confirm that our method selects more diverse molecules regarding molecular properties. Furthermore, although our method does not explicitly use structure-based descriptors (e.g., ECFP and MACCS key), it successfully selects diverse molecules in terms of MPD values calculated with the Tanimoto coefficient of such structure-based descriptors. We also validate the practical effectiveness of our method via experiments on out-of-domain settings, where we use datasets in different domains between training of GNNs and selection of molecules.

## Method

**Figure 1:**
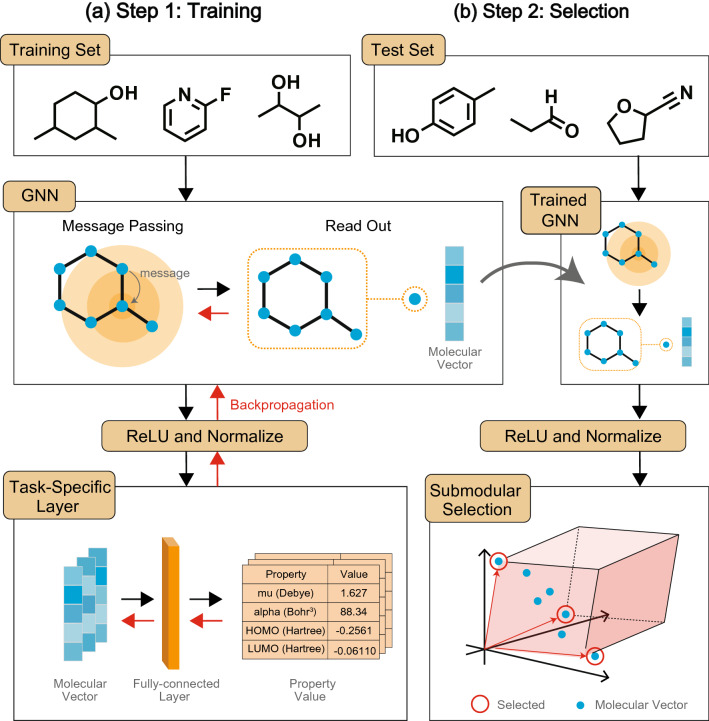
A high-level sketch of our method. In Step 1, a GNN is trained with property prediction tasks. The black and red arrows indicate the forward pass and backpropagation, respectively. In Step 2, the trained GNN is used to generate molecular vectors of candidate molecules, and then molecules are selected via submodular function maximization.

This section presents our molecular selection method, which comprises two steps: training a GNN that generates molecular vectors and selecting GNN-based molecular vectors via submodular function maximization. Figure 1 shows a high-level sketch of our method. In Step 1, we train a GNN and task-specific layers with property prediction tasks, where the GNN converts molecular graphs into molecular vectors and the task-specific layers take them as input and predict molecular properties. In this step, parameters of the GNN and the task-specific layers are updated by the error backpropagation method. In Step 2, we transform graphs of candidate molecules into molecular vectors by using the GNN trained in Step 1, and then select a predetermined number of molecular vectors based on submodular function maximization.

We also introduce a new property-based diversity criterion, which quantifies the diversity of selected molecules as the Wasserstein distance to uniform distributions defined on molecular property values. Intuitively, we regard a set of molecules as diverse if the property values of those molecules are evenly distributed.

### Graph neural networks for generating molecular vectors

We briefly explain how GNNs generate molecular vectors. GNNs are deep learning architectures that work on graph domains. Taking a graph with node and edge features as input, GNNs capture structures of the graph by iteratively passing messages, which are calculated based on the features. Specifically, each node iteratively receives messages from its neighbors, aggregates them, and pass them to its neighbors; after this message passing phase, a molecular vector, denoted by ${\varvec{x}}$, is computed based on the resulting messages of all nodes. Along the way, messages are updated with certain parameterized functions. Our specific choice of a GNN architecture is Attentive FP^41^, which is reported to achieve high performances in molecular property prediction. For the sake of completeness, we present mathematical details of GNNs in the “Supplementary information”.

In the task-specific layer, molecular properties, ${\varvec{y}}$, are predicted with molecular vector ${\varvec{x}}$ via simple linear regression as $\hat{{\varvec{y}}} = {\varvec{W}} {\varvec{x}}+ {\varvec{b}}$, where $\hat{{\varvec{y}}}$ is a prediction of ${\varvec{y}}$. In the training step (Step 1 in Fig. 1), we update ${\varvec{W}}$, ${\varvec{b}}$, and the parameters of the GNN by backpropagation, where a loss function is the mean squared error between $\hat{{\varvec{y}}}$ and ${\varvec{y}}$. Consequently, the GNN, which captures structures of molecular graphs via message passing, is trained to predict molecular properties. Therefore, the trained GNN generates molecular vectors taking both structures and properties of molecules into account.

### Selection of diverse molecular vectors

Given molecular vectors generated by the trained GNN, we aim to obtain a set of diverse molecules by selecting diverse molecular vectors. For selecting diverse vectors, we utilize the mathematical framework called submodular function maximization.

#### Submodular function maximization

Submodular function maximization has been studied in the field of combinatorial optimization. This framework enables development of effective diverse selection methods by offering flexible models for representing the diversity and efficient selection algorithms with mathematical performance guarantees; below we detail these two advantages.

The first advantage of using the submodular-function-maximization framework is that there are various known functions for representing the diversity. To find a diverse subset from a large pool of molecules, researchers specify a diversity criterion and search for a diverse subset based on the criterion. Here, a diversity criterion is formally regarded as a set function, which assigns to each subset a real value that indicates how diverse the subset is. Some of such functions have a special property called *submodularity*, and they are called *submodular functions*. Many submodular functions have been developed as diversity criteria for various kinds of data such as images, documents, and videos. Therefore, we can choose a suitable function from them for modeling the diversity of molecular vectors. For example, the Shannon entropy is known to satisfy submodularity with respect to the selection of random variables. Other diversity criteria that have submodularity include the ROUGE-N score for document summarization^51,52^ and facility location functions^53^. In the area of bioinformatics, submodular functions for peptide identification are also developed^54^.

The second advantage of the submodular-function-maximization framework is that we can utilize various simple, efficient, and mathematically rigorous algorithms for selecting a diverse subset. When selecting a subset from a large number of molecular vectors, there are exponentially many possible candidate subsets. Therefore, we need efficient algorithms for finding diverse subsets. In a series of studies on submodular function maximization, many simple and efficient algorithms for finding subsets with large submodular function values have been developed. Notably, the resulting submodular function values are often guaranteed to be nearly optimal by mathematical analyses. Therefore, once we specify a submodular function as a diversity criterion, we can automatically ensure that those algorithms return highly diverse subsets with respect to the criterion. Among such algorithms, the greedy algorithm is widely used due to its simplicity, efficiency, and strong mathematical guarantee^44^.

In the “Supplementary information”, we present mathematical details of submodular function maximization and the greedy algorithm.

#### Log-determinant function

**Figure 2:**
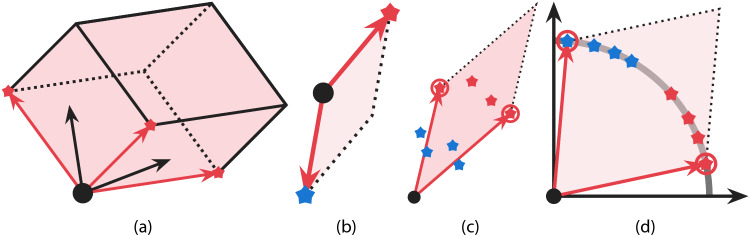
Graphical explanation of (**a**) the log-determinant function, (**b**) the effect of ReLU, and (**c** and **d**) the effect of normalization. In all figures above, the black dot indicates the origin. (**a**) is a parallelotope spanned by vectors colored in red. (**b**) illustrates an example where the log-determinant function value for dissimilar vectors becomes small if vectors are allowed to have negative elements. Here and in the next two figures, points with different colors (red and blue) represent molecules with dissimilar properties, while those with the same colors have similar properties. (**c**) shows why maximizing the log-determinant function without normalization may result in a non-diverse selection, and (**d**) presents how normalization helps the log-determinant function maximization to select diverse vectors. Note that (**d**) is generally different from the normalized version of (**c**), i.e., vectors generated by the GNN with normalization are different from those obtained by normalizing vectors generated by the GNN without normalization. This is because the backpropagation is performed through the normalization layer, and thus the presence of the normalization layer affects how the GNN parameters are updated. As a result, the GNN is trained to generate molecular vectors so that the task-specific layer can predict molecular properties based on the angles of vectors, as in (**d**).

In our computational experiments, we use a submodular function called a *log-determinant function*, which quantifies the diversity of selected molecular vectors based on the volume of a parallelotope spanned by the selected vectors. As depicted in Fig. 2a, the more diverse the directions of vectors are, the larger the volume of the parallelotope spanned by them. Thus the log-determinant function provides a volume-based measure of the diversity of vectors, and it is often used for expressing the diversity of vector datasets^55^. Note that the volume-based diversity can capture relationships of vectors that cannot be represented in a pairwise manner. Therefore, the log-determinant function yields a different selection rule than existing methods such as MaxSum and MaxMin, which use pairwise dissimilarities of molecular structures.

Formally, the log-determinant function is defined as follows. Suppose that *n* candidate molecules are numbered by $1,\ldots ,n$ and that the *i*th molecule is associated with *m*-dimensional molecular vector ${\varvec{x}}_i$ for $i=1,\ldots ,n$. Let ${\varvec{X}}=[{\varvec{x}}_1\ {\varvec{x}}_2\ \ldots \ {\varvec{x}}_n]$ be an $m \times n$ matrix whose columns are given by *n* molecular vectors. For any $S\subseteq N{:}{=} \{1,\ldots ,n\}$, we denote by ${\varvec{X}}[S]$ an $m \times |S|$ submatrix of ${\varvec{X}}$ with columns restricted to *S*. We define the log-determinant function $f_{\text {logdet}}$ by

$$\begin{aligned} f_{\text {logdet}}(S) = \log \det ({\varvec{X}}[S]^\top {\varvec{X}}[S] + {\mathbf {I}}_{|S|}) \end{aligned}$$

for any $S \subseteq N$, where ${\mathbf {I}}_{|S|}$ is the $|S| \times |S|$ identity matrix. The relationship between the $f_{\text {logdet}}$ value and the volume of a parallelotope can be formally described as follows. Let $\tilde{{\varvec{x}}_i}$ ($i=1,\ldots ,n$) be a vector of length $m+n$ such that the first *m* elements are given by ${\varvec{x}}_i$, the ($m+i$)-th element is 1, and the others are 0. When $S\subseteq N$ is selected, $f_{\text {logdet}}(S)$ indicates the volume of a parallelotope spanned by $\{\tilde{{\varvec{x}}}_i\}_{i\in S}$.

Given the function, $f_{\text {logdet}}$, and the number, $k$, of molecules to be selected, the greedy algorithm operates as follows: it first sets $S = \emptyset$ and sequentially adds $i \in N\setminus S$ with the largest $f_{\text {logdet}}(S\cup \{i\}) - f_{\text {logdet}}(S)$ value to *S* while $|S| < k$ holds. In our computational experiments, we use a fast implementation of the greedy algorithm specialized for the log-determinant function^56^. Function $f_{\text {logdet}}$ satisfies $f_{\text {logdet}}(\emptyset ) = 0$, monotonicity (i.e., $S\subseteq T$ implies $f_{\text {logdet}}(S) \le f_{\text {logdet}}(T)$), and submodularity. With these properties, we can mathematically guarantee that the greedy algorithm returns a subset whose $f_{\text {logdet}}$ value is at least $1-1/{\text {e}}\approx 63\%$ of an optimal selection.

### Refinements to molecular vector generation: ReLU and normalization

We refine the GNN-based vector generation process so that it works better with the log-determinant function. Specifically, we make GNNs output non-negative and normalized. Below we detail why we need these refinements and explain how to modify the vector generation process.

First, as in Fig. 2b, if vectors are allowed to have negative elements, nearly origin-symmetric vectors form a parallelotope with a small volume even though their directions are diverse. Consequently, the log-determinant function fails to indicate that such molecular vectors correspond to diverse molecules. To circumvent this issue, we add a ReLU layer to the end of the GNN, which makes all entries of output vectors non-negative.

Second, if GNNs are allowed to output vectors with different norms, task-specific layers may distinguish molecules with different properties based on the norm of molecular vectors. In such cases, maximizing the log-determinant function may result in selecting non-diverse vectors due to the following reason. As mentioned above, the log-determinant function represents the volume of the parallelotope spanned by selected vectors, and the volume becomes larger if selected vectors have larger norms. Consequently, molecular vectors with larger norms are more likely to be selected, which may result in selecting molecules with almost the same properties as in Fig. 2c. To resolve this problem, after passing through the ReLU layer, we normalize molecular vectors so that their norms become 1 by projecting them onto a hypersphere. In other words, we add a normalization layer that transforms molecular vector ${\varvec{x}}$ as

$$\begin{aligned} \hat{{\varvec{x}}} = \frac{{\varvec{x}}}{\Vert {\varvec{x}}\Vert }. \end{aligned}$$

As a result, $\hat{{\varvec{x}}}$ becomes non-negative and its norm is equal to 1. In the training phase, we train the GNN with the additional ReLU and normalization layers, where non-negative normalized vector $\hat{{\varvec{x}}}$ is used for predicting property values as $\hat{{\varvec{y}}} = {\varvec{W}} \hat{{\varvec{x}}} + {\varvec{b}}$. Due to the above normalization, the task-specific layers cannot distinguish molecular vectors by using their norms, and thus the GNN learns to generate molecular vectors so that task-specific layers can predict molecular property values based not on norms but on angles of vectors. Consequently, as illustrated in Fig. 2d, diverse molecular vectors can be obtained by maximizing the log-determinant function value. We experimentally confirmed that GNNs trained with normalization yield similar prediction results to those obtained without normalization (see, the “Supplementary information”). This implies that GNNs trained with normalization can successfully generate molecular vectors whose angles have enough information for predicting molecular properties.

### Property-based evaluation of diversity

By using our selection method, we can select molecules so that corresponding molecular vectors are diverse. However, even if molecular vectors are diverse, selected molecules themselves may not be diverse. This issue is also the case with the existing structure-based methods, and it has been overlooked in previous studies. That is, the existing methods select molecules that are diverse in terms of the Tanimoto coefficient of molecular descriptors (e.g., MACCS keys or ECFP), and thus those methods naturally achieve high mean pairwise distance (MPD) values, which are also calculated by using the Tanimoto coefficient of such descriptors. If we are to evaluate selection methods fairly, we need diversity criteria that do not use molecular descriptors employed by selection methods. This section presents such a criterion for evaluating the diversity of selected molecules in terms of their property values without using molecular vectors. In contrast to the existing structure-based criteria (e.g., the aforementioned MPD values), our criterion is based on the diversity of property values, thus offering a new perspective for evaluating the diversity of molecules.

Our idea is to regard molecular property values as diverse if evenly distributed over an interval on the property-value line. We quantify this notion of the diversity using a statistical distance between a distribution of property values of selected molecules and a uniform distribution. As a distance between two distributions, we use the Wasserstein distance, which is defined by the minimum cost of transporting the mass of one distribution to another, as detailed below. We call this diversity criterion the Wasserstein distance to a uniform distribution (WDUD). A smaller WDUD value implies that selected molecules are more diverse since the distribution of their property values is closer to being uniform.

Formally, WDUD is defined as follows. Let $v_{\max }$ and $v_{\min }$ be the maximum and minimum property values, respectively, in a given list of molecules. Suppose that $k$ molecules with property values $y_1,y_2,\ldots ,y_k$ are selected from the list. We assign probability mass $1/k$ to each $y_i$ and compute how far this discrete distribution is from a uniform distribution over $[v_{\min }, v_{\max }]$. Let *V* and *U* be the cumulative distribution functions of the two distributions, respectively. Defining the transportation cost from $y\in [v_{\min }, v_{\max }]$ to $y_i$ as $|y - y_i|$, the WDUD value can be computed as $\int _{v_{\min }}^{v_{\max }} |U(x) - V(x)|{\text {d}}x$^50^, which we use for quantifying the diversity of property values $\{y_1,y_2,\ldots ,y_k\}$ of selected molecules.

**Figure 3:**
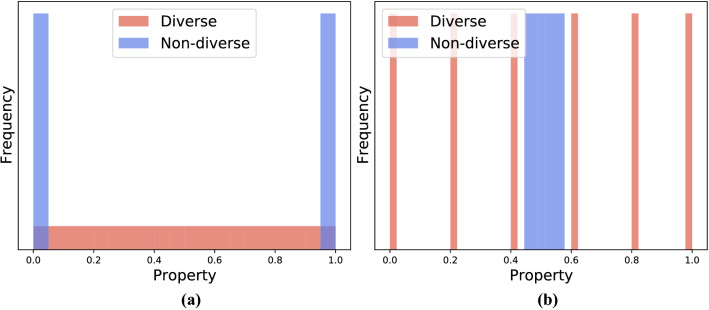
Graphical explanation of why the (**a**) variance and (**b**) Kullback–Leibler (KL) divergence are inappropriate. In (**a**), the variance of a diverse set (red) is smaller than that of a non-diverse set (blue), which does not suit our idea of diversity. In (**b**), the KL divergence of a diverse set (red) is equal to that of a non-diverse set (blue).

There are other possible choices of statistical distances, such as the variance or the Kullback–Leibler (KL) divergence. However, the Wasserstein distance is more suitable for measuring the diversity than the variance and the KL divergence for the following reasons. If we use the variance of property values of selected molecules as a diversity measure, a set of molecules with extreme property values is regarded as diverse, although this selection is biased since it ignores property values nearby the mean (see, Fig. 3a). If we use the KL divergence between the property-value distribution of selected molecules and the uniform distribution, the distance structure of the support is ignored unlike WDUD, which takes the $\ell _1$-distance, $|y - y_i|$, into account. As a result, we cannot distinguish molecular sets with completely different diversities as in Fig. 3b.

### Wasserstein greedy: a property-based benchmark method

In the computational experiments, we use a benchmark method that is intended to minimize the WDUD value directly. To the best of our knowledge, selecting a set of molecules that exactly minimizes the WDUD value reduces to mixed integer programming, which is computationally hard in general. Instead, we select molecules with small WDUD values by using a simple greedy heuristic, which starts with the empty set and repeatedly selects a molecule that yields the largest decrease in the WDUD value. When considering the WDUD of multiple molecular properties, we normalize the property values to [0, 1] and use the mean WDUD value. In the experiments, property values are known only for a training dataset, while we have to select molecules from a test dataset. Therefore, we compute WDUD values by using property values predicted by the trained GNN (without the normalization technique). Compared with our proposed method, this benchmark method is specialized for achieving small WDUD values (i.e., diversity of molecular property values), while it does not explicitly use information on molecular structures.

### Existing structure-based selection methods and evaluation criterion

We also use MaxMin and MaxSum as baseline methods, which greedily select molecules based on dissimilarities of molecular descriptors. We use MACCS keys and ECFP as descriptors and define the dissimilarity of those descriptors based on the Tanimoto coefficient, i.e., given the *i*th and *j*th descriptors, we compute the Tanimoto similarity of them and subtract it from 1 to obtain dissimilarity values $d_{i,j}$. Given dissimilarity values $d_{i,j}$, MaxSum and MaxMin operate as with the greedy algorithm; formally, MaxMin (resp. MaxSum) sequentially adds $i\in N\setminus S$ with the largest $\min _{j\in S} d_{i,j}$ (resp. $\sum _{j\in S} d_{i,j}$) value to *S* whlie $|S| < k$ holds, where the first molecule $i\in N$ is set to the one with the largest $\sum _{j\ne i}d_{i,j}$ value. We denote MaxMin and MaxSum methods by MM and MS, respectively, and MACCS keys and ECFP by MK and EF, respectively, for short. We use, for example, MS-MK to represent the MaxSum method that uses MACCS-keys as descriptors.

When evaluating selection methods in the experiments, we also use the mean pairwise dissimilarity (MPD), the existing structure-based criterion, in addition to WDUD. Specifically, given dissimilarity values $d_{i,j}$ for all pairs in *n* molecules, we compute an MPD value as $$\frac{1}{\left( {\begin{array}{c}n\\ 2\end{array}}\right) } \sum _{i < j} d_{i,j}$$. We define the dissimilarity values by the Tanimoto dissimilarity of MACCS keys or ECFP. Depending on the choice of descriptors, we denote the diversity criterion by MPD-MK or MPD-EF, respectively.

### Details of computaitonal experiments

We conducted computational experiments with the QM9 dataset in MoleculeNet^57,58^, which is a quantum mechanical dataset with labels of energetic, electronic, and thermodynamic properties computed based on the density functional theory (DFT). Each molecule in the dataset is associated with 12 property values: dipole moment in Debye (mu), isotropic polarizability in ${\hbox {Bohr}}^{3}$ (alpha), highest occupied molecular orbital energy in Hartree (HOMO), lowest unoccupied molecular orbital energy in Hartree (LUMO), gap between HOMO and LUMO in Hartree (gap), electronic spatial extent in ${\hbox {Bohr}}^{2}$ (R2), zero-point vibrational energy in Hartree (ZPVE), internal energy at 0 K in Hartree (U0), internal energy at 298.15 K in Hartree (U), enthalpy at 298.15 K in Hartree (H), free energy at 298.15 K in Hartree (G), and heat capacity at 298.15 K in cal/(mol K) (Cv). Following the previous work^41^, we used all the 12 properties to train GNNs. The QM9 dataset contains 133,885 molecules, and we randomly divided them into three datasets as is done in the previous study^41^: 80% (107,108 molecules) for training a GNN, 10% (13389 molecules) for validating prediction accuracy of the trained GNN, and 10% (13388 molecules) for a test dataset, from which we selected molecules. Each method selected 133 molecules (1% of the test data) from the test data. Note that when training GNNs, we did not use the test data. We thus created the situation where we select molecules whose property values are unknown in advance.

The diversity of property values of selected molecules was evaluated by computing WDUD values for each molecular property. In this evaluation, we used the above 12 properties in the QM9 dataset. However, among the 12 properties, the use of U0, U, H, and G would be inappropriate for evaluating the chemical diversity because their magnitudes depend mostly on the system size. For example, these values are more similar between acetone and acetamide, isoelectronic molecules, than between acetone and methyl–ethyl–ketone, even though most chemists would say that acetone and methyl–ethyl–ketone are both alkyl ketones and chemically more similar. Therefore, we additionally used molecular energy values divided by the number of electrons (denoted by $N_{\text{{elec}}}$) in the evaluation to weaken the system-size dependence and focus more on chemical diversity. These values for U0, U, H, and G are denoted by U0/$N_{\text{{elec}}}$, U/$N_{\text{{elec}}}$, G/$N_{\text{{elec}}}$, and H/$N_{\text{{elec}}}$, respectively. Similarly, we used variants of the two values, ZPVE and Cv, divided by $N_{\text{{mode}}} = 3N_{\text{{atom}}} - 6$, where $N_{\text{{atom}}}$ is the number of atoms. These values for ZPVE and Cv are denoted by ZPVE/$N_{\text{{mode}}}$ and Cv/$N_{\text{{mode}}}$, respectively. Consequently, for evaluating molecular diversity based on WDUD values, we used 18 properties in total: the 12 properties of the QM9 dataset and the additional six properties, ZPVE/$N_{\text{{mode}}}$, U0/$N_{\text{{elec}}}$, U/$N_{\text{{elec}}}$, G/$N_{\text{{elec}}}$, H/$N_{\text{{elec}}}$, and Cv/$N_{\text{{mode}}}$.

We also conducted computational experiments on the out-of-domain setting. That is, while the GNN is trained with the QM9 dataset, we select molecules from other test datasets than QM9, where we know nothing about the target property labels. This setting is more challenging than the previous one since the test datasets are completely different from QM9; in particular, the target property labels are different from the aforementioned 12 properties of QM9. On the other hand, this setting is more realistic since GNNs are usually trained on some large datasets, while we often want to select molecules from new test datasets that belong to other domains than those of training datasets. In the experiments, we used three test datasets obtained from MoleculeNet: the Delaney dataset (ESOL)^59^, the free solvation database (FreeSolv)^60^, and the lipophilicity dataset (Lipop)^61^. ESOL contains 1128 molecules labeled by log-scale water solubility in mol/L. FreeSolv contains 642 molecules labeled by experimentally measured hydration free energy in water in kcal/mol. Lipop contains 4200 molecules labeled by experimentally measured octanol/water distribution coefficient (logD). These property labels were used only when computing WDUD values for evaluation. For each of the three datasets, we selected 100 molecules and evaluated their diversity. Note that unlike the previous case, we select molecules without knowing what properties are used when evaluating WDUD values. Thus, this setting models a situation where we want to select molecules that are diverse regarding some unknown properties.

## Results and discussion

We present the results obtained by the following molecular selection methods:

- **SubMo-GNN** (*Sub*modularity-based *Mo*lecular selection with *GNN*-based molecular vectors) is our proposed method, which greedily maximizes the log-determinant function^55^ defined with GNN-based molecular vectors.
- **WG-GNN** (*W*asserstein *G*reedy with *GNN*-based prediction) is our new benchmark method. It selects molecules by greedily minimizing the WDUD values, where molecular property values are predicted by the trained GNN.
- **MS-MK** is the existing MaxSum algorithm^33^ that uses MACCS keys^24^ as molecular descriptors.
- **MM-MK** is the existing MaxMin algorithm^34,35^ that uses MACCS keys^24^ as molecular descriptors.
- **MS-EF** is the existing MaxSum algorithm^33^ that uses ECFP^23^ as molecular descriptors.
- **MM-EF** is the existing MaxMin algorithm^34,35^ that uses ECFP^23^ as molecular descriptors.
- **Random** selects molecules randomly according to the distribution of a test dataset.

We briefly mention the position of each method. WG-GNN is a benchmark method that is specialized for the diversity of property values, while the structure-based baseline methods, MS-MK, MM-MK, MS-EF, and MM-EF, focus on the diversity of molecular structures. Our SubMo-GNN is an intermediate of the two kinds of methods and can leverage information of both molecular structures and properties, since the GNN-based molecular vectors are generated by taking molecular graphs as input and training the GNN with property-prediction tasks.

### Property-based diversity evaluation with WDUD

We evaluated the diversity of property values of selected molecules by the Wasserstein distance to uniform distribution (WDUD). Note that a smaller WDUD value is better since it means the distribution of selected molecules is closer to being uniform.

**Figure 4:**
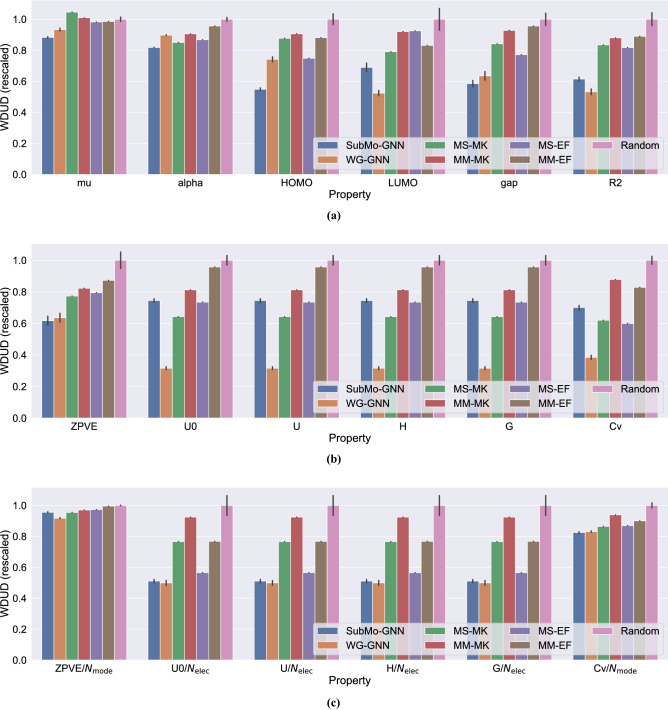
Bar charts of WDUD values. All the values are rescaled so that those of Random become 1. (**a**) Shows the results on the first six properties. (**b**) is the results on the six properties correlated with $N_{\text{{elec}}}$ or $N_{\text{{mode}}}$ (the WDUD values are computed with the raw property values). (**c**) is a modified version of (**b**), where the property values are divided by $N_{\text{{elec}}}$ or $N_{\text{{mode}}}$ for making those values capture subtle molecular characteristics such as connectivity patterns.

Table 1 shows the WDUD values attained by the six methods for the aforementioned 18 properties. Since the results of SubMo-GNN and WG-GNN fluctuate due to the randomness caused when training GNNs, we performed five independent runs and calculated the mean and standard deviation. The results of Random also vary from trial to trial, and thus we present the mean and standard deviation of five independent trials. Figure 4 visualizes the results in Table 1, where the WDUD values are rescaled so that those of Random become 1 for ease of comparison.

**Table 1:** WDUD values for each property in QM9 dataset. Since SubMo-GNN, WG-GNN, and Random have randomness, their results are shown by means and standard deviations over five trials.

	SUBMO-GNN	WG-GNN	MS-MK	MM-MK	MS-EF	MM-EF	Random
mu (Debye)	${ 10.65}\ {{\pm }}\ {0.06}$	$11.26 \pm 0.12$	12.62	12.18	11.85	11.89	$12.06 \pm 0.19$
alpha (Bohr${}^3$)	${ 37.03}\ {{\pm }}\ {0.07}$	$40.65 \pm 0.13$	38.50	41.01	39.30	43.30	$45.28 \pm 0.60$
HOMO (Hartree)	${ 0.02786}\ {{\pm }}\ {0.00049}$	$0.03764 \pm 0.00091$	0.04452	0.04599	0.03797	0.04473	$0.05075 \pm 0.00197$
LUMO (Hartree)	$0.02905 \pm 0.00133$	${ 0.02208}\ {{\pm }}\ {0.00079}$	0.03331	0.03879	0.03898	0.03502	$0.04213 \pm 0.00329$
gap (Hartree)	${ 0.03541}\ {{\pm }}\ {0.00138}$	$0.03844 \pm 0.00186$	0.05092	0.05614	0.04668	0.05788	$0.06051 \pm 0.00264$
R2 (Bohr${}^2$)	$359.0 \pm 7.8$	${ 310.9}\ {{\pm }}\ {12.1}$	487.3	513.8	477.3	519.5	$583.9 \pm 27.4$
ZPVE (Hartree)	${ 0.02320}\ {{\pm }}\ {0.00118}$	$0.02391 \pm 0.00120$	0.02907	0.03093	0.02986	0.03282	$0.03760 \pm 0.00219$
U0 (Hartree)	$85.09 \pm 1.47$	${ 36.14}\ {{\pm }}\ {1.14}$	73.39	92.80	83.92	109.4	$114.2 \pm 3.8$
U (Hartree)	$85.09 \pm 1.47$	${ 36.13}\ {{\pm }}\ {1.14}$	73.39	92.80	83.92	109.3	$114.2 \pm 3.8$
H (Hartree)	$85.09 \pm 1.47$	${ 36.13}\ {{\pm }}\ {1.14}$	73.39	92.80	83.92	109.3	$114.2 \pm 3.8$
G (Hartree)	$85.10 \pm 1.47$	${ 36.14}\ {{\pm }}\ {1.14}$	73.39	92.81	83.93	109.4	$114.2 \pm 3.8$
Cv (cal/(mol K))	$5.593 \pm 0.129$	${ 3.072}\ {{\pm }}\ {0.118}$	4.950	7.011	4.785	6.613	$7.984 \pm 0.226$
ZPVE/$N_{\text{mode}}$ (Hartree)	$0.001533 \pm 0.000006$	${ 0.001472}\ {{\pm }}\ {0.000007}$	0.001531	0.001557	0.001561	0.001598	$0.001604 \pm 0.000004$
U0/$N_{\text{elec}}$ (Hartree)	$0.3848 \pm 0.0087$	${ 0.3757}\ {{\pm }}\ {0.0129}$	0.5770	0.6961	0.4257	0.5780	$0.7529 \pm 0.0536$
U/$N_{\text{elec}}$ (Hartree)	$0.3848 \pm 0.0087$	${ 0.3757}\ {{\pm }}\ {0.0129}$	0.5770	0.6961	0.4257	0.5780	$0.7529 \pm 0.0536$
H/$N_{\text{elec}}$ (Hartree)	$0.3848 \pm 0.0087$	${ 0.3757}\ {{\pm }}\ {0.0129}$	0.5770	0.6961	0.4257	0.5780	$0.7529 \pm 0.0536$
G/$N_{\text{elec}}$ (Hartree)	$0.3848 \pm 0.0087$	${ 0.3757}\ {{\pm }}\ {0.0129}$	0.5770	0.6961	0.4257	0.5780	$0.7529 \pm 0.0536$
Cv/$N_{\text{mode}}$ (cal/(mol K))	${ 0.4681}\ {{\pm }}\ {0.0031}$	$0.4715 \pm 0.0031$	0.4905	0.5328	0.4936	0.5109	$0.5674 \pm 0.0106$

In this experiment, each method obtains a single set of molecules, for which we calculate the 18 WDUD values. Therefore, choosing a set of molecules that attains small WDUD values for some properties may result in large WDUD values for other properties. Such a choice of molecules does not meet our purpose, and it is better to balance the trade-off so that none of the 18 WDUD values become too large. A reasonable way to check whether this is achieved is to compare the results with those of Random. If WDUD values of some properties become larger than those of Random, it is probable that selected molecules are biased; that is, the diversity of some properties is sacrificed for achieving small WDUD values of other properties. On the other hand, WG-GNN is expected to achieve almost the best WDUD values since it aims to minimize WDUD values directly (this, however, can result in non-diverse selection regarding other aspects than WDUD, as we will discuss later). Therefore, we below discuss the results regarding the WDUD values of WG-GNN as benchmarks that are close to the best possible ones.

We first discuss the results of SubMo-GNN and the existing structure-based methods in comparison with Random and WG-GNN. Figure 4 shows that SubMo-GNN, MS-EF, and MM-EF attained smaller WDUD values than Random for all molecular properties. This indicates that both our method and the ECFP-based methods were able to choose diverse molecules in terms of WDUD, even though they do not explicitly minimize WDUD. Since we did not feed the test dataset when training GNNs, the results suggest that GNNs well generalized to unknown molecules and achieved diverse selection from the test dataset consisting of unknown molecules. In contrast to SubMo-GNN and the ECFP-based methods, MS-MK and MM-MK resulted in larger WDUD values in mu than Random as in Fig. 4a. That is, the selection methods based on MACCS keys failed to select diverse molecules with respect to mu values. This suggests that selection methods that use only structural information can sometimes result in non-diverse selection in terms of molecular property values. On the other hand, as expected, WG-GNN achieved the smallest WDUD values in 12 out of the 18 properties. Surprisingly, however, SubMo-GNN achieved better WDUD values than WG-GNN in six properties, demonstrating the effectiveness of SubMo-GNN for selecting molecules with diverse property values.

We then compare our SubMo-GNN with the existing structure-based selection methods. Compared to MaxMin-based methods (MM-MK and MM-EF), SubMo-GNN achieved smaller WDUD values for all properties. SubMo-GNN also outperformed MaxSum-based methods (MS-MK and MS-EF) for all but six properties (U0, U, H, G, Cv, and ZPVE/$N_{\text{{mode}}}$). Note that U0, U, H, and G are related to molecular energies and their values are strongly correlated with each other; previous studies have reported that property prediction methods applied to the QM9 dataset exhibited almost the same performances as regards the four properties^41^. This is consistent with our results in Fig. 4b, where each method attained almost the same performance regarding the four properties. Furthermore, when the energy-related properties are divided by $N_{\text{{elec}}}$, MS-MK and MS-EF are outperformed by SubMo-GNN (see the results on U0/$N_{\text{{elec}}}$, U/$N_{\text{elec}}$, H/$N_{\text{{elec}}}$, and G/$N_{\text{{elec}}}$ in Fig. 4c). In view of this, the MaxSum-based methods seem to have put too much weight on the diversity of properties correlated with $N_{\text{{elec}}}$, which resulted in biased selections and degraded the WDUD values of mu. In summary, in terms of WDUD values, the overall performance of SubMo-GNN is better than those of the existing structure-based methods.

**Figure 5:**
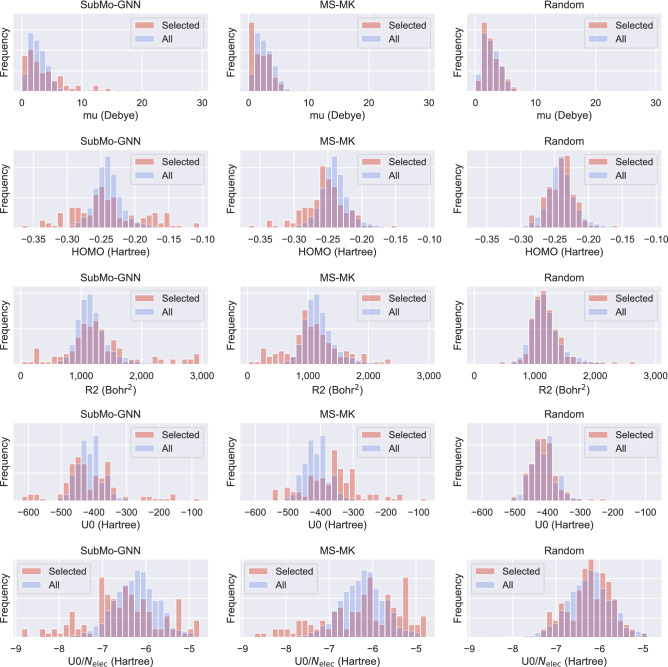
Property-value distributions of selected molecules (red) and all molecules in the test dataset (blue). The horizontal axis indicates property values, which are partitioned into 30 bins. We let the height show the probability density for ease of comparison. Results of the same property and method are aligned in a row and a column, respectively.

Figure 5 shows property-value distributions of all molecules in the dataset (blue) and molecules selected by each method (red). The horizontal and vertical axes represent property values and frequency, respectively. For ease of comparison, the histogram height is normalized to indicate the density rather than the count. We regard a set of molecules as diverse if its distribution is close to being uniform. As expected, the distribution of molecules selected by Random is close to the distribution of the original dataset. By contrast, SubMo-GNN and MS-MK selected molecules that did not appear so frequently in the dataset, particularly for HOMO, R2, U0, and U0/$N_{\text{{elec}}}$. As a result, the distributions of selected molecules became closer to being uniform than Random. Regarding the results of mu, both SubMo-GNN and MS-MK chose many molecules with near-zero mu values; this seems to be necessary for selecting diverse molecules regarding other properties than mu due to the aforementioned trade-off between properties. Nevertheless, MS-MK chose too many molecules with near-zero mu values, resulting in a biased distribution. This visually explains why the WDUD value of MS-MK in mu is larger than that of Random. Compared with MS-MK, SubMo-GNN selected more molecules with large mu values, which alleviated the bias and led to diverse selection in all properties. SubMo-GNN selected more molecules with large R2 and high HOMO values than MS-MK, and consequently SubMo-GNN ’s distributions were closer to being uniform. In U0, however, MS-MK selected more molecules with high U0 values than SubMo-GNN and MS-MK ’s distribution was closer to being uniform than SubMo-GNN. By contrast, as regards U0/$N_{\text{elec}}$, MS-MK selected too many molecules with high $N_{\text{elec}}$ values compared with SubMo-GNN, resulting in a distribution that is farther from being uniform.

To conclude, by incorporating supervised learning of GNNs into the system of diverse molecular selection, our method can select diverse molecules regarding target molecular properties in the sense that their distributions are close to being uniform. On the other hand, if we use standard molecular descriptors (e.g., MACCS keys and ECFP) that encode only structural information of molecules, selected molecules can be non-diverse regarding some molecular properties.

### Structure-based diversity evaluation with MPD

We then evaluated selection methods in terms of the diversity of molecular substructures. As a criterion for evaluating the diversity of molecular substructures, we used the mean pairwise dissimilarity (MPD), where molecular descriptors were given by MACCS keys or ECFP. We denote those criteria by MPD-MK and MPD-EF for short. A larger MPD value is better since it implies that selected molecules are more dissimilar to each other. It should be noted that MS-MK and MS-EF greedily maximize MPD-MK and MDP-EF, respectively, and thus they are inherently advantageous in this setting. MM-MK and MM-EF also explicitly maximize the diversity calculated with MACCS keys and ECFP, respectively, and thus this setting is also favorable for them. By contrast, SubMo-GNN and WG-GNN use neither MACCS keys nor ECFP, and thus it has no inherent advantage as opposed to the structure-based methods.

Table 2 shows the results. As expected, MS-MK and MM-MK, which explicitly aim to maximize the diversity calculated with MACCS keys, achieved high MPD-MK values. In particular, MS-MK attained a far higher MPD-MK value than the others. This result is natural since MS-MK has the inherent advantage of greedily maximizing MPD-MK. As regards MPD-EF, all methods achieved high MPD values. Note that although SubMo-GNN and WG-GNN used neither MACCS keys nor ECFP, they attained higher MPD values than Random and performed comparably to (sometimes outperformed) the structure-based methods. These results imply that selecting molecules with diverse property values is helpful in selecting molecules with diverse structures.

**Table 2:** MPD values with MACCS keys and ECFP. For SubMo-GNN, WG-GNN, and Random, means and standard deviations over five trials are shown.

	SUBMO-GNN	WG-GNN	MS-MK	MM-MK	MS-EF	MM-EF	Random
MPD-MK	$$0.8423 \pm 0.0029$$	$$0.8536 \pm 0.0018$$	$$ { 0.9102}$$	0.8592	0.8565	0.7924	$$0.7734 \pm 0.0129$$
MPD-EF	$$0.9518 \pm 0.0017$$	$$0.9435 \pm 0.0006$$	0.9558	0.9427	$$ { 0.9733}$$	0.9520	$$0.9247 \pm 0.0063$$

At this point, the effectiveness of selecting molecules with diverse predicted property values has been confirmed for the case where GNNs are trained on a training QM9 dataset and molecules are selected from a test QM9 dataset, i.e., training and test datasets are in the same domain. In practice, however, we often encounter a situation where GNNs are trained on some large datasets, while we select molecules from new datasets whose domains are different from those of training datasets. In such cases, the diversity of properties registered in the training datasets does not always imply the diversity of molecules in test datasets. Below we experimentally study such out-of-domain settings.

### Experiments on out-of-domain setting

We performed experiments on the out-of-domain setting. Specifically, while we trained GNNs on the QM9 dataset as with the previous section, we selected molecules from other test datasets: ESOL, FreeSolv, and Lipop. SubMo-GNN used molecular vectors generated by the trained GNN, and WG-GNN selected molecules greedily to minimize WDUD values of the QM9 properties predicted by using the trained GNN. Note that we cannot train GNNs to predict ESOL, FreeSolv, and Lipop values since nothing about those properties is available. In other words, we consider training GNNs without knowing that they are used for selecting diverse molecules from ESOL, FreeSolv, and Lipop datasets. On the other hand, the structure-based descriptors, ECFP and MACCS keys, have nothing to do with the property labels of test datasets. Therefore, the existing structure-based methods select molecules in the same way as in the previous section. Unlike the previous QM9 case, we selected 100 molecules independently for each of ESOL, FreeSolv, and Lipop since the three datasets consist of different molecules.

In this setting, since target property labels and structures of molecules in test datasets are unavailable in advance, we want to select diverse molecules regarding a wide variety of unknown molecular characteristics. To this end, selection methods should not overfit to certain molecular characteristics; they should select molecules that are diverse regarding various aspects, including both property values and structures.

**Figure 6:**
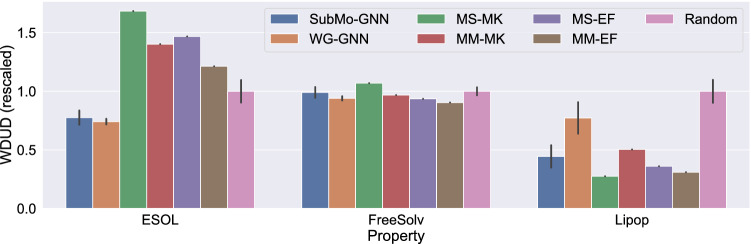
Bar charts of WDUD values in the out-of-domain setting. All the values are rescaled so that those of Random become 1.

Table 3 shows the WDUD values achieved by each method for ESOL, FreeSolv, and Lipop, and Fig. 6 visualizes the results. SubMo-GNN and WG-GNN selected molecules more diversely than Random, even though the GNN was feeded no information on ESOL, FreeSolv, and Lipop. From the fact that WG-GNN achieved small WDUD values, we can say that molecules with diverse ESOL, FreeSolv, and Lipop values can be obtained by selecting molecules with diverse QM9 property values. On the other hand, although the structure-based methods achieved small WDUD values for FreeSolv and Lipop, they selected less diverse molecules than Random in ESOL. This implies that, as with the case of mu values in the QM9 dataset, structure-based methods can result in non-diverse selection regarding some property values.

**Table 3:** WDUD values in the out-of-domain setting. For SubMo-GNN, WG-GNN, and Random, means and standard deviations over five trials are shown.

	SUBMO-GNN	WG-GNN	MS-MK	MM-MK	MS-EF	MM-EF	Random
ESOL	$0.7544 \pm 0.0663$	${ 0.7213 \pm 0.0260}$	1.638	1.364	1.428	1.180	$0.9726 \pm 0.1046$
FreeSolv	$$2.180 \pm 0.113$$	$$2.069 \pm 0.045$$	2.354	2.130	2.060	$$ { 1.991}$$	$$2.201 \pm 0.084$$
Lipop	$$0.3077 \pm 0.0739$$	$$0.5349 \pm 0.1040$$	$$ { 0.1906}$$	0.3488	0.2497	0.2145	$$0.6922 \pm 0.0758$$

**Figure 7:**
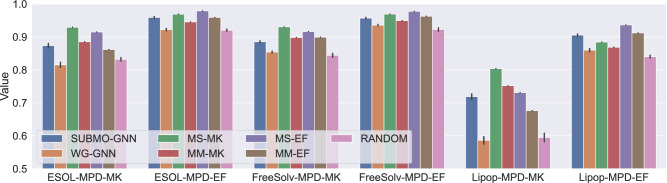
Bar charts of MPD values in the out-of-domain setting.

Table 4 and Fig. 7 present MPD-MK and MPD-EF values for each dataset. SubMo-GNN achieved higher MPD values in all cases than WG-GNN and Random, and it performed comparably to the structure-based methods. On the other hand, WG-GNN failed to outperform Random in ESOL-MPD-MK and Lipop-MPD-MK. These results suggest that WG-GNN does not always perform well regarding the diversity of structures in the out-of-domain setting. By contrast, the results of SubMo-GNN imply that the GNN-based molecular vectors learned on the QM9 dataset well generalize to out-of-domain datasets and successfully convey information on both molecular properties and structures, thus enabling SubMo-GNN to select diverse molecules regarding both properties and structures even in the out-of-domain setting.

**Table 4:** MPD values in the out-of-domain setting. For SubMo-GNN, WG-GNN, and Random, means and standard deviations over five trials are shown.

	SUBMO-GNN	WG-GNN	MS-MK	MM-MK	MS-EF	MM-EF	Random
ESOL-MPD-MK	$$0.8736 \pm 0.0074$$	$$0.8153 \pm 0.0087$$	$$ { 0.9293}$$	0.8854	0.9149	0.8617	$$0.8321 \pm 0.0061$$
ESOL-MPD-EF	$$0.9590 \pm 0.0032$$	$$0.9222 \pm 0.0041$$	0.9690	0.9455	$$ { 0.9791}$$	0.9590	$$0.9204 \pm 0.0036$$
FreeSolv-MPD-MK	$$0.8853 \pm 0.0033$$	$$0.8541 \pm 0.0034$$	$$ { 0.9305}$$	0.8982	0.9162	0.8991	$$0.8442 \pm 0.0066$$
FreeSolv-MPD-EF	$$0.9570 \pm 0.0030$$	$$0.9357 \pm 0.0028$$	0.9693	0.9494	$$ { 0.9775}$$	0.9627	$$0.9226 \pm 0.0056$$
Lipop-MPD-MK	$$0.7188 \pm 0.0097$$	$$0.5860 \pm 0.0127$$	$$ { 0.8038}$$	0.7519	0.7310	0.6764	$$0.5949 \pm 0.0144$$
Lipop-MPD-EF	$$0.9056 \pm 0.0038$$	$$0.8600 \pm 0.0054$$	0.8843	0.8686	$$ { 0.9362}$$	0.9119	$$0.8401 \pm 0.0048$$

Note that in the above QM9 and out-of-domain experiments, only SubMo-GNN achieved better performances than Random in all criteria. This suggests that the proposed combination of the log-determinant function maximization and the GNN-based descriptors, which are designed to represent both molecular properties and structures, is effective for delivering stable performances in diverse molecular selection regarding various aspects of molecules.

### Discussion on MaxSum and MaxMin with GNN vectors and effects of normalization

The previous experiments confirmed that GNN-based molecular vectors can capture both properties and structures of molecules, which enabled our SubMo-GNN to select diverse molecules. In this additional experiment, we again use the QM9 training and test datasets and present an ablation study to see how the choice of selection methods affects outputs if GNN-based molecular vectors are used as descriptors. Moreover, as an attempt to elucidate how the black-box GNN-based vector generation affects the molecular selection phase, we take a closer look at norms of molecular vectors generated by GNNs and examine how the normalization procedure changes the behavior of selection methods.

In this section, all selection methods use GNN-based molecular vectors, and thus we denote our SubMo-GNN simply by SubMo. We use the three selection methods: SubMo, MaxSum (MS), and MaxMin (MM). Each method employs GNN-based molecular vectors with and without normalization, denoted by “w/N” and “w/o N”, respectively, as molecular descriptors. Regarding MaxSum and MaxMin, the pairwise dissimilarity between two vectors is given by their Euclidian distance.

Table 5 presents WDUD values achieved by each method. SubMo and MS tended to achieve smaller WDUD values than MM. It also shows that normalization did not always yield better WDUD values. Only from the results of WDUD values, it may seem that MaxSum without normalization (MS w/o N) performs as well as (or better than) SubMo w/ and w/o N. As discussed below, however, the superiority of MS w/o N is brittle and it can result in non-diverse selection in some cases.

**Table 5:** WDUD values in the ablation study. Since the GNN-based vector-generation process has randomness, the results of all methods are shown by means and standard deviation over five trials.

	SUBMO w/o N	SUBMO w/ N	MS w/o N
mu (Debye)	${ 10.47}\ {{\pm }}\ {0.10}$	$10.65 \pm 0.06$	$11.88 \pm 0.06$
alpha (Bohr${}^3$)	$38.76 \pm 0.47$	$37.03 \pm 0.07$	$37.64 \pm 0.49$
HOMO (Hartree)	$0.02931 \pm 0.00194$	${ 0.02786 \pm 0.00049}$	$0.04135 \pm 0.00115$
LUMO (Hartree)	${ 0.02243}\ {{\pm }}\ {0.00212}$	$0.02905 \pm 0.00133$	$0.03360 \pm 0.00058$
gap (Hartree)	$0.03542 \pm 0.00181$	${ 0.03541}\ {{\pm }}\ {0.00138}$	$0.05428 \pm 0.00086$
R2 (Bohr${}^2$)	$373.4 \pm 18.6$	$359.0 \pm 7.8$	${ 211.2}\ {{\pm }}\ {4.2}$
ZPVE (Hartree)	$0.02800 \pm 0.00125$	$0.02320 \pm 0.00118$	${ 0.01542}\ {{\pm }}\ {0.00097}$
U0 (Hartree)	$93.39 \pm 2.08$	$85.09 \pm 1.47$	${ 68.82}\ {{\pm }}\ {0.62}$
U (Hartree)	$93.39 \pm 2.08$	$85.09 \pm 1.47$	${ 68.82}\ {{\pm }}\ {0.62}$
H (Hartree)	$93.39 \pm 2.08$	$85.09 \pm 1.47$	${ 68.82}\ {{\pm }}\ {0.62}$
G (Hartree)	$93.40 \pm 2.08$	$85.10 \pm 1.47$	${ 68.83}\ {{\pm }}\ {0.62}$
Cv (cal/(mol K))	$5.788 \pm 0.155$	$5.593 \pm 0.129$	${ 4.893}\ {{\pm }}\ {0.091}$
ZPVE/$N_{\text{mode}}$ (Hartree)	$0.001572 \pm 0.000008$	$0.001533 \pm 0.000006$	$0.001547 \pm 0.000004$
U0/$N_{\text{elec}}$ (Hartree)	$0.3956 \pm 0.0293$	$0.3848 \pm 0.0087$	${ 0.2163}\ {{\pm }}\ {0.0139}$
U/$N_{\text{elec}}$ (Hartree)	$0.3956 \pm 0.0293$	$0.3848 \pm 0.0087$	${ 0.2163}\ {{\pm }}\ {0.0139}$
H/$N_{\text{elec}}$ (Hartree)	$0.3956 \pm 0.0293$	$0.3848 \pm 0.0087$	${ 0.2163}\ {{\pm }}\ {0.0139}$
G/$N_{\text{elec}}$ (Hartree)	$0.3955 \pm 0.0293$	$0.3848 \pm 0.0087$	${ 0.2163}\ {{\pm }}\ {0.0139}$
Cv/$N_{\text{mode}}$ (cal/(mol K))	${ 0.4419}\ {{\pm }}\ {0.0068}$	$0.4681 \pm 0.0031$	$0.4521 \pm 0.0016$

	MS w/ N	MM w/o N	MM w/ N
mu (Debye)	$11.21 \pm 0.21$	$11.15 \pm 0.14$	$10.69 \pm 0.18$
alpha (Bohr${}^3$)	${ 36.63}\ {{\pm }}\ {0.36}$	$38.86 \pm 0.59$	$39.05 \pm 0.73$
HOMO (Hartree)	$0.03101 \pm 0.00128$	$0.03375 \pm 0.00147$	$0.03067 \pm 0.00100$
LUMO (Hartree)	$0.03385 \pm 0.00228$	$0.02422 \pm 0.00046$	$0.03140 \pm 0.00151$
gap (Hartree)	$0.04084 \pm 0.00300$	$0.04108 \pm 0.00159$	$0.04061 \pm 0.00267$
R2 (Bohr${}^2$)	$316.2 \pm 20.9$	$342.0 \pm 15.3$	$361.9 \pm 12.6$
ZPVE (Hartree)	$0.01677 \pm 0.00088$	$0.02501 \pm 0.00176$	$0.02896 \pm 0.00076$
U0 (Hartree)	$71.93 \pm 2.06$	$89.49 \pm 2.91$	$87.40 \pm 2.20$
U (Hartree)	$71.93 \pm 2.06$	$89.49 \pm 2.91$	$87.40 \pm 2.20$
H (Hartree)	$71.93 \pm 2.06$	$89.49 \pm 2.91$	$87.40 \pm 2.20$
G (Hartree)	$71.94 \pm 2.06$	$89.50 \pm 2.91$	$87.40 \pm 2.20$
Cv (cal/(mol K))	$5.299 \pm 0.135$	$6.124 \pm 0.110$	$5.877 \pm 0.132$
ZPVE/$N_{\text{mode}}$ (Hartree)	${ 0.001497}\ {{\pm }}\ {0.000006}$	$0.001565 \pm 0.000011$	$0.001553 \pm 0.000006$
U0/$N_{\text{elec}}$ (Hartree)	$0.3487 \pm 0.0407$	$0.3785 \pm 0.0194$	$0.4525 \pm 0.0141$
U/$N_{\text{elec}}$ (Hartree)	$0.3487 \pm 0.0407$	$0.3785 \pm 0.0194$	$0.4525 \pm 0.0141$
H/$N_{\text{elec}}$ (Hartree)	$0.3487 \pm 0.0407$	$0.3785 \pm 0.0194$	$0.4525 \pm 0.0141$
G/$N_{\text{elec}}$ (Hartree)	$0.3487 \pm 0.0407$	$0.3784 \pm 0.0194$	$0.4525 \pm 0.0141$
Cv/$N_{\text{mode}}$ (cal/(mol K))	$0.4812 \pm 0.0013$	$0.4632 \pm 0.0051$	$0.4777 \pm 0.0036$

**Figure 8:**
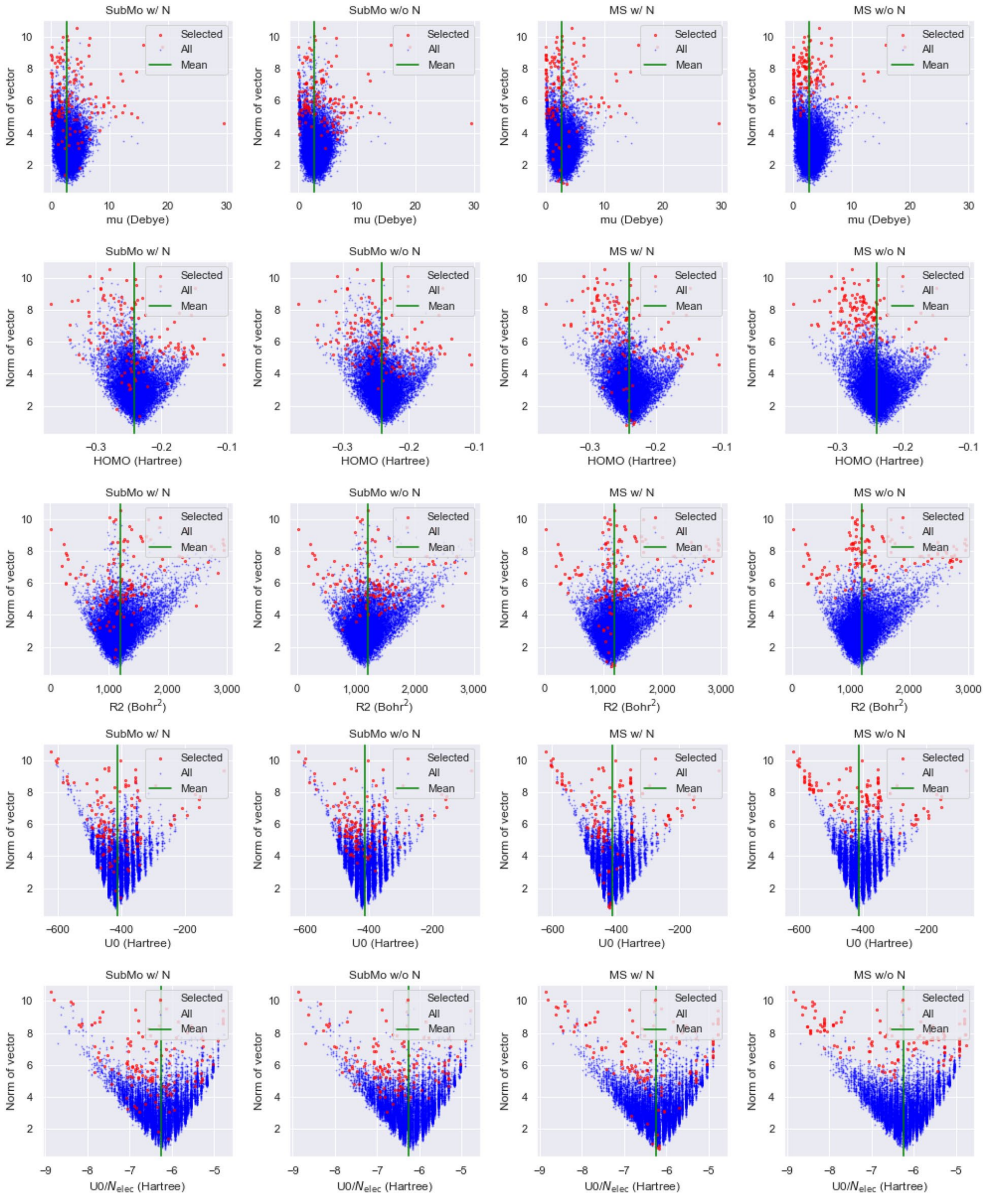
Scatter plots of norms vs. property values. Blue points correspond to all molecules in the test dataset, and red points indicate molecules selected by each method.

Figure 8 illustrates the relationship between property values and the norms of molecular vectors generated by a GNN without normalization. The vertical and horizontal axes indicate norms and property values, respectively. The blue and red points correspond to all molecules in the test dataset and selected molecules, respectively. The green vertical lines show the means of property values in the test dataset. The figures imply the correlation between the norm and deviation of property values from their means. That is, GNNs tend to assign large norms to molecules whose property values are far from the means, and molecules with small norms tend to have property values that are close to the means. This tendency suggests that GNNs convey the information of how far molecular property values are from the means by using norms of molecular vectors.

Since MS greedily maximizes the sum of pairwise dissimilarity values, it prefers selecting molecular vectors that are distant to each other. As a result, MS tend to select molecular vectors with large norms, as we can confirm in the rightmost column of Fig. 8. In the case of the QM9 dataset, GNNs assigned large norms to some molecules whose property values were close the means. Therefore, by simply selecting molecules with large norms as MS did, molecules with diverse property values can be obtained. However, depending on datasets and how GNNs are trained, the correlation between norms and property values can become much stronger. In such cases, MS cannot select molecules whose property values are close to the means, resulting in biased selection.

Compared with MS, SubMo selected molecular vectors with various norms. Therefore, even if norms and property values are strongly correlated, SubMo is expected to select molecules with more diverse property values than MS. As regards normalization, norms of vectors selected by SubMo w/ N were almost the same as those selected by SubMo w/o N, while there is a clear difference between MS w/ N and MS w/o N.

To conclude, no single selection method outperforms in all cases, and thus we should employ appropriate selection methods that are suitable for datasets at hand. Nevertheless, MaxSum seems to rely too much on norms of molecular vectors relative to SubMo, and thus we are required to carefully examine molecular vectors when using MaxSum. We finally emphasize that a notable advantage of SubMo is its theoretical guarantee. That is, the log-determinant function values achieved by the greedy algorithm is always at least 63% of optimal function values.

### Detailed experimental settings and running times

We trained Attentive FP with the following hyperparameters: radius = 2, T = 2, fingerprint dimension = 280, dropout = 0.5, weight decay = 0, learning rate = 0.0004, and epoch = 300, where radius and T are the number of times the hidden states are updated in the message passing and readout phases, respectively. In the QM9 experiment, the size of the matrix ${\varvec{X}}$ in the log-determinant function is $13388\times 280$ (the number of candidates $\times$ the dimension of molecular vectors). In the ESOL, FreeSolv, and Lipop experiments, the sizes of ${\varvec{X}}$ are $1128\times 280$, $642\times 280$, and $4200\times 280$, respectively.

We performed computational experiments on Amazon EC2 P3.2xlarge. It has a single Tesla V100 GPU (16GB) and 8 vCPUs (61GB). In the QM9 experiments, training of GNN took 2900 seconds. For selecting molecules, SubMo-GNN, WG-GNN, MS-MK, MM-MK, MS-EF, and MM-EF took 5.1, 5700, 240, 240, 200, and 200 s, respectively. In the ESOL experiments, SubMo-GNN, WG-GNN, MS-MK, MM-MK, MS-EF, and MM-EF took 0.56, 650, 1.7, 1.7, 1.3, and 1.3 s, respectively. In the FreeSolv experiments, they took 0.51, 420, 0.57, 0.57, 0.40, and 0.40 s, respectively, in the same order. In the Lipop experiments, they took 0.58, 2600, 26, 26, 35, and 36 s, respectively, in the same order. Note that while the greedy algorithm in SubMo-GNN used a specialized implementation technique^56^, the other algorithms are implemented in a naive manner and thus have room for acceleration. Therefore, the presented running times are only for reference. Faster implementation of the baseline algorithms is beyond the scope of this paper.

## Conclusion

We addressed the problem of selecting diverse molecules for facilitating chemical space exploration. Our method consists of two steps: construction of molecular vectors using the GNN and selection of molecules via maximizing submodular functions defined with molecular vectors. Owing to the use of GNNs trained with property prediction tasks, we can take both molecular structures and properties into account for selecting diverse molecules. Moreover, the submodular function maximization framework enables the greedy algorithm to return subsets of molecules that are mathematically guaranteed to be nearly optimal. We also introduced a new evaluation criterion, the Wasserstein distance to uniform distributions (WDUD), to measure the diversity of sets of molecules based on property values. Computational experiments on the QM9 dataset showed that our method could successfully select diverse molecules as regards property values. Regarding the diversity of molecular structures, it performed comparably to the existing structure-based methods (MaxSum and MaxMin with MACCS keys and ECFP). Experiments with out-of-domain settings demonstrated that our method with the GNN trained on the QM9 dataset could select molecules with diverse property values and structures from out-of-domain datasets: ESOL, FreeSolv, and Lipop. To conclude, our diverse selection method can help researchers efficiently explore the chemical space, which will bring great advances in searching for novel chemical compounds and reactions.

We finally mention some future directions. In this study, we evaluated the diversity of molecular properties using the 12 properties of the QM9 dataset, ESOL, FreeSolv, and Lipop. On the other hand, molecular properties used in medicinal chemistry, e.g., Pharmacokinetic properties (logP), drug-likeness (QED), and biological activities, are important in the field of virtual screening. Although the goal of diverse selection is different from that of virtual screening, evaluating diverse selection methods based on properties such as logP and QED may offer an interesting direction of study. Mathematically, studying the relationship between the log-determinant function value and the WDUD value is interesting future work.

## Supplementary Information

Supplementary Information.

## Data Availability

Source codes of our method are available at https://github.com/tomotomonakanaka/SUBMO.git, which were implemented in Python 3.7.10. We converted SMILES into MACCS key, ECFP, and molecular graphs by using RDKit 2018.09.1, which is available at https://www.rdkit.org/. The QM9 dataset was downloaded from Xiong’s GitHub repository (https://github.com/OpenDrugAI/AttentiveFP). The ESOL, FreeSolv, and Lipop datasets were downloaded through DeepChem^62^. GNNs were implemented using PyTorch 1.8.0^63^, DGL 0.5.3^64^, and DGL-LifeSci 0.2.6 (available at https://github.com/awslabs/dgl-lifesci).
