# Supplementary material for: Selecting molecules with diverse structures and properties by maximizing submodular functions of descriptors learned with graph neural networks
Source: Sci Rep. 2022 Jan 21;12:1124. doi: 10.1038/s41598-022-04967-9 (PMC8782878; doi:10.1038/s41598-022-04967-9)
Supplement: Supplementary file 1 — Supplementary Information. [file 41598_2022_4967_MOESM1_ESM.pdf]

Supplementary Information

Selecting Molecules with Diverse Structures and Properties by Maximizing Submodular Functions of Descriptors Learned with Graph Neural Networks

Tomohiro Nakamura, Shinsaku Sakaue, Kaito Fujii, Yu Harabuchi, Satoshi Maeda, and Satoru Iwata

Mathematical details of GNNs

We present mathematical details of GNNs based on a simple message passing neural network (MPNN), which provides a general framework for dealing with supervised learning tasks on graph-structured datasets.[1] Let G be an undirected graph with node set $V(G)$ and edge set $E(G)$. When transforming molecular graphs into vectors, nodes and edges correspond to atoms and bonds, respectively. Suppose that node features $\{\boldsymbol{h}_v^0 | v \in V(G)\}$ and edge features $\{\boldsymbol{c}_{vu} | vu \in E(G)\}$ are assigned to the nodes and edges. The forward pass of an MPNN is divided into two phases: the message passing phase and the readout phase. In the message passing phase, message $\boldsymbol{m}_v^t$ and hidden state $\boldsymbol{h}_v^t$ of each node $v \in V$ are alternately updated for $t = 1, \ldots, T$ as follows:

$$\boldsymbol{m}_v^t = \sum_{u \in N(v)} M_t(\boldsymbol{h}_v^{t-1}, \boldsymbol{h}_u^{t-1}, \boldsymbol{c}_{vu}) \quad \text{and} \quad \boldsymbol{h}_v^t = U_t(\boldsymbol{h}_v^{t-1}, \boldsymbol{m}_v^t),$$

where M_t is a message function, U_t is an update function, and $N(v)$ represents neighbors of v. After the T-th iteration, we apply readout function R to hidden-state vectors for computing vector $\boldsymbol{x}$ of graph G as follows:

$$\boldsymbol{x} = R(\{\boldsymbol{h}_v^T | v \in V(G)\}).$$

In the training step, parameters of M_t, U_t, and R, together with those of task-specific layers, are updated by backpropagation.

With our specific choice of a GNN architecture, called Attentive FP,[2] atom features are updated by using a graph attention mechanism in the message-passing phase.[3] Then it updates a feature of a virtual node, which connects all atoms to represent the whole molecular graph, in the readout phase by using the same embedding attention mechanism. Our method uses the updated vector of the virtual node as molecular vector $\boldsymbol{x}$.

Mathematical details of submodular function maximization

We present mathematical details of submodular function maximization. We consider a situation where n items are given, from which we select diverse k items. We number the items by $1, \ldots, n$ and consider selecting an index subset $S \subseteq N := \{1, \ldots, n\}$.

Let $f : 2^N \to \mathbb{R}$ be a set function, which assigns diversity value $f(S)$ to every subset $S \subseteq N$ of items. Function f is said to be submodular if

$$f(S) + f(T) \geq f(S \cap T) + f(S \cup T)$$

holds for every pair of $S, T \subseteq N$. The problem of selecting up to k items that maximizes submodular function f can be written as

$$\text{maximize} \quad f(S) \qquad \text{subject to} \quad S \subseteq N, \ |S| \leq k.$$

A standard algorithm for this problem is the greedy algorithm. The algorithm first sets $S = \emptyset$ and then sequentially adds to S an element, $i \in N$, that yields the largest increase in the f value. If f satisfies $f(\emptyset) = 0$, monotonicity (i.e., $S \subseteq T$ implies $f(S) \leq f(T)$), and submodularity, we can mathematically guarantee that the greedy algorithm returns subset $S \subseteq N$ such that $f(S)$ is at least $1 - 1/\mathrm{e} \approx 63\%$ of the optimal value, i.e., $\max_{S \subseteq N : |S| \leq k} f(S)$.[4]

Results on property prediction tasks

We experimentally studied how the use of the normalization layer affects molecular property prediction. With the QM9 dataset, we trained GNNs with and without normalization via property prediction tasks. Table 1 presents mean absolute error (MAE) values achieved by GNNs trained with and without normalization. We see that the performances are similar to each other, even though vectors generated by the GNN with normalization loses the norm information. This implies that, when the GNN is trained with normalization, the information that should be retained in the norm is represented by the angles of vectors. Therefore, the resulting molecular vectors are expected to work well with the log-determinant function, as explained in the method section.

Table 1. MAE values on QM9 achieved by using GNNs with and without normalization. The results are shown by means and standard deviation over 5 trials.

	w/o normalization	w/ normalization
mu (Debye)	0.4352 ± 0.0072	0.4265 ± 0.0060
alpha (Bohr^3)	0.5895 ± 0.0204	0.5741 ± 0.0253
HOMO (Hartree)	0.003596 ± 0.000042	0.003477 ± 0.000032
LUMO (Hartree)	0.004095 ± 0.000083	0.003993 ± 0.000094
gap (Hartree)	0.005108 ± 0.000085	0.005013 ± 0.000079
R2 (Bohr^2)	31.12 ± 1.19	29.71 ± 0.82
ZPVE (Hartree)	0.001815 ± 0.000092	0.001786 ± 0.000076
U0 (Hartree)	1.186 ± 0.175	1.334 ± 0.192
U (Hartree)	1.186 ± 0.175	1.334 ± 0.192
H (Hartree)	1.186 ± 0.175	1.334 ± 0.192
G (Hartree)	1.186 ± 0.175	1.334 ± 0.192
Cv (cal/(mol K))	0.3044 ± 0.0264	0.2844 ± 0.0152

References

1. Gilmer, J., Schoenholz, S. S., Riley, P. F., Vinyals, O. & Dahl, G. E. Neural message passing for quantum chemistry. In *Proceedings of the 34th International Conference on Machine Learning*, vol. 70, 1263–1272 (2017).

2. Xiong, Z. *et al.* Pushing the boundaries of molecular representation for drug discovery with the graph attention mechanism. *J. Med. Chem.* **63**, 8749–8760 (2020).

3. Veličković, P. *et al.* Graph attention networks. In *Proceedings of the 18th International Conference on Learning Representations* (2018).

4. Nemhauser, G. L., Wolsey, L. A. & Fisher, M. L. An analysis of approximations for maximizing submodular set functions—I. *Math. Program.* **14**, 265–294 (1978).
